# Closed-loop neuromodulation: a paradigm shift in precision neuroscience for clinical and cognitive applications

**DOI:** 10.3389/fnins.2026.1943905

**Published:** 2026-09-22

**Authors:** Hongliang Lu, Danming Miao

**Affiliations:** Department of Military Medical Psychology, Air Force Medical University, Xi’an, China

**Keywords:** brain biomarker, closed-loop neuromodulation, cognitive regulation, neurological and psychiatric disorders, precision neurostimulation

## Abstract

Closed-loop neuromodulation systems are critically synthesized here with respect to their foundational principles, mechanistic underpinnings, and systematic classifications. This comprehensive survey covers their rapid research progress and expanding clinical applications, while rigorously exploring their emerging potential for cognitive augmentation. Concurrently, persistent challenges and future research directions are identified. The scope encompasses invasive, non-invasive, and peripheral techniques, with careful examination of their therapeutic utility in neurological and psychiatric disorders—including epilepsy, major depression, chronic pain syndromes, and movement disorders—alongside exploratory applications in cognitive enhancement among healthy individuals. Enabling technologies, such as advanced wireless biosensors, sophisticated AI-driven adaptive algorithms, and novel stimulation modalities, are critically assessed, juxtaposed with inherent challenges like robust biomarker validation, long-term device stability, and complex ethical considerations. Closed-loop neuromodulation systems have demonstrably achieved superior efficacy, enhanced personalization, and improved safety profiles compared to conventional open-loop paradigms. The accelerating pace of technological innovation is rapidly translating these advances from bench to bedside. This technology signifies a fundamental evolution in personalized medicine and offers substantial potential for cognitive optimization, necessitating concerted interdisciplinary collaboration to surmount extant challenges and ensure successful, responsible implementation.

## 1 Introduction

The ability to modulate neural activity has become a cornerstone of modern neuroscience and clinical practice, offering therapeutic avenues for conditions once deemed intractable (Ashkan et al., 2017). Techniques ranging from invasive deep brain stimulation (DBS) to non-invasive transcranial magnetic stimulation (TMS) are predicated on the principle of neuroplasticity—the brain’s inherent capacity to reorganize its structure and function in response to stimuli (Cramer et al., 2011; Jayathilake et al., 2025; Kricheldorff et al., 2022; Yuan et al., 2020). Historically, these interventions have operated in an “open-loop” fashion, delivering stimulation according to a fixed set of parameters (e.g., frequency, intensity, duration) prescribed by a clinician (Guidotti et al., 2025; Kukanti et al., 2026; Zhang H. et al., 2025). While effective for many patients, this approach overlooks the highly dynamic and individualized nature of brain function (Miniussi and Bortoletto, 2025; Yang et al., 2021; Jin et al., 2024). The brain is not a static entity; its activity fluctuates from moment to moment (Damoiseaux et al., 2006; Wen et al., 2021), and the optimal parameters for neuromodulation can vary significantly across individuals and even within the same individual over time (Van Hoornweder et al., 2025; Soleimani et al., 2023). This variability can lead to suboptimal therapeutic outcomes, undesirable side effects, and the development of tolerance (Guerra et al., 2020; Plow et al., 2012).

To address these limitations, the field is rapidly transitioning towards closed-loop neuromodulation, also known as adaptive, responsive, or smart neuromodulation (Wansbrough et al., 2024). This paradigm establishes a bidirectional, real-time dialogue with the nervous system. A closed-loop system continuously monitors a specific neural or physiological biomarker (Zhou et al., 2019; Zhou et al., 2018), uses a control algorithm to interpret this signal in the context of a desired therapeutic state, and dynamically adjusts stimulation parameters to maintain that state (Green and Chakravarthy, 2025; Zanos, 2019; Scangos et al., 2021). This approach promises a new era of personalized and precise medicine, where interventions are tailored to the unique and fluctuating neurophysiology of each individual (Ciampi de Andrade and Shirvalkar, 2025).

The growing interest in this field is reflected in recent publication trends (Li et al., 2022). An analysis of keywords such as “Closed-loop transcranial modulation” and “closed-loop stimulation” using Stork’s Big Analysis software (https://www.storkapp.me/meta/) reveals a significant increase in publications, with a projected 50% year-over-year growth rate and a notable concentration of papers in 2025 and beyond. This surge is driven by converging advancements in materials science, microelectronics, computational neuroscience, and artificial intelligence (AI). Comprehensive reviews have recently synthesized the burgeoning landscape of BCI-based neural recording and stimulation technologies, highlighting their translational promise for neuropsychiatric disorders (Xu et al., 2026).

In this review, the terms “adaptive,” “responsive,” and “closed-loop” neuromodulation are used interchangeably to refer to systems that incorporate real-time feedback for stimulation adjustment. Unless otherwise specified, “closed-loop” is used as the preferred term throughout.

This review aims to provide a comprehensive overview of the state of the art in closed-loop neuromodulation. We will first delineate the fundamental principles and mechanisms that define these systems and classify the diverse range of technologies employed. We will then survey the major research progress and clinical applications across neurological, psychiatric, and rehabilitative domains. Subsequently, we will explore the exciting potential for closed-loop systems to be used for cognitive enhancement in healthy individuals. Finally, we will discuss the enabling technologies, future directions, and the critical challenges—both technical and ethical—that must be addressed to realize the full transformative potential of this powerful approach (Figure 1).

**Figure 1:**
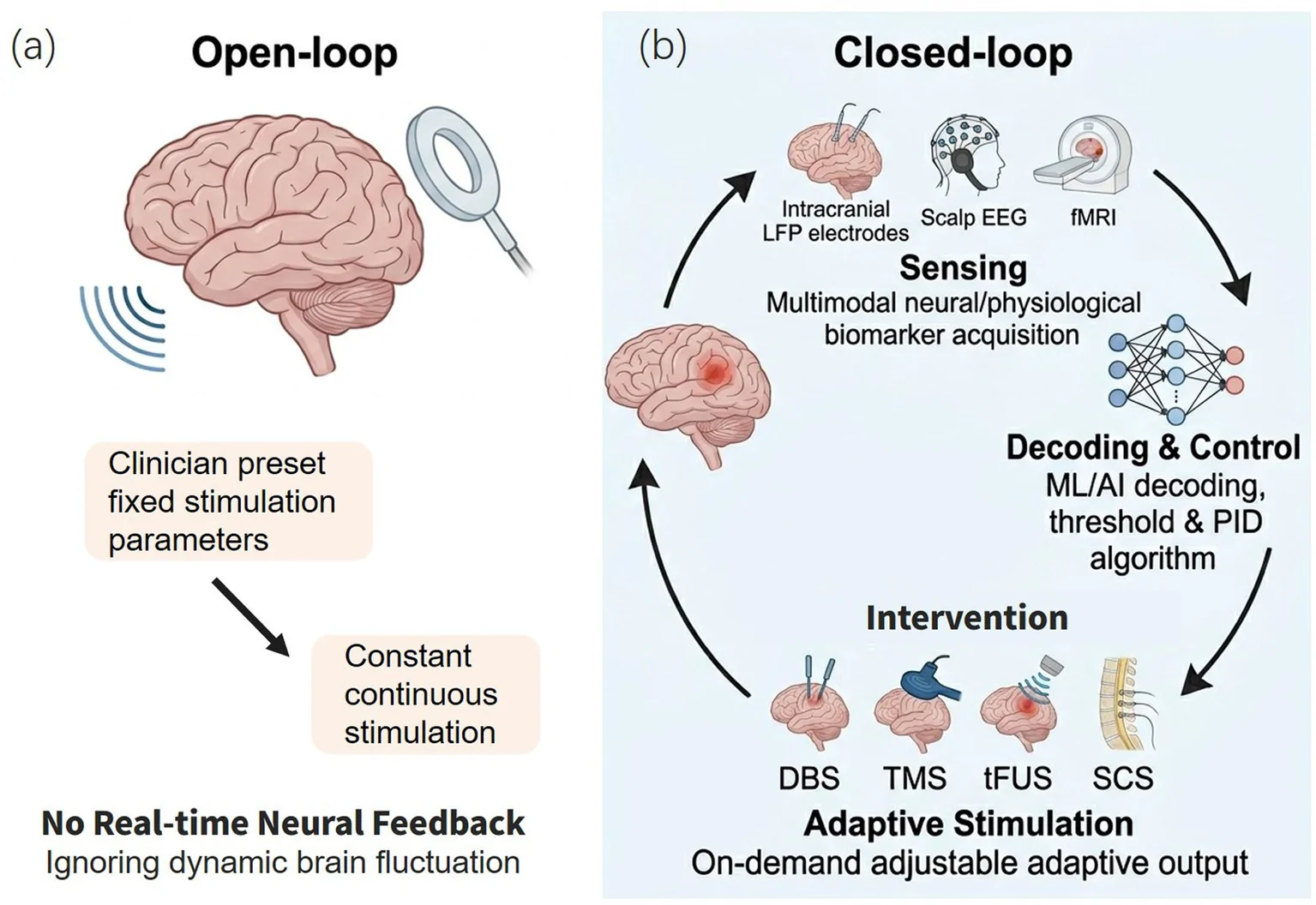
Schematic comparison of open-loop and closed-loop neuromodulation workflow. **(a)** Conventional open-loop neuromodulation delivers constant stimulation via fixed pre-set parameters without real-time feedback of dynamic brain activity. **(b)** Closed-loop architecture constructs a bidirectional cyclic feedback loop composed of four functionally distinct modules: real-time multimodal neural/physiological signal sensing (left), neural-state decoding (upper middle, typically using AI/ML algorithms), control decision-making (lower middle, using threshold, PID, or model-predictive controllers), and dynamically adjusted on-demand adaptive stimulation output (right). The distinction between decoding (state estimation) and control (policy generation) is emphasized to reflect current engineering practice (Wang et al., 2025; Chu et al., 2025). The schematic was designed to illustrate that feedback latency and biomarker specificity are the two critical performance determinants in closed-loop systems.

## 2 Core closed-loop mechanisms

The defining feature of a closed-loop neuromodulation system is its ability to create a feedback circuit that links brain state to therapeutic intervention. This circuit is universally composed of three essential components: sensing, decoding, and stimulation.

### 2.1 Sensing and monitoring

The first step is the continuous acquisition of biological signals that reflect the state of the target neural system. The choice of signal depends on the specific application, balancing factors like invasiveness, spatial and temporal resolution, and signal-to-noise ratio. Invasive methods provide high-fidelity signals from deep brain structures, including local field potentials (LFPs) and single-neuron recordings, which can reveal highly specific neural activity patterns (Pang et al., 2026). Non-invasive techniques such as electroencephalography (EEG) offer excellent temporal resolution for tracking brain oscillations from the scalp (Chen H. et al., 2025), while functional magnetic resonance imaging (fMRI) can provide whole-brain coverage, albeit with lower temporal resolution (Soleimani et al., 2023). Multimodal data fusion approaches can combine the strengths of different modalities to create a more comprehensive picture of brain function (Zhang et al., 2020). Beyond neural signals, physiological biomarkers like heart rate, skin conductance, or body temperature can also serve as inputs for the control loop (Ouyang et al., 2023).

### 2.2 Decoding and control

The raw data acquired during the sensing phase must be processed and interpreted in real-time to identify a “control variable” or “biomarker” that signifies a specific brain state (e.g., an impending seizure, a depressive state, a moment of memory consolidation). This decoding step is computationally intensive and increasingly relies on sophisticated algorithms, particularly from the fields of machine learning (ML) and AI (Chen et al., 2022; Kahlon et al., 2026). The control algorithm then compares the current state to a desired target state and determines the necessary adjustments to the stimulation parameters. This can be a simple threshold-based trigger (e.g., stimulate when beta-band power exceeds a certain level) or a more complex proportional-integral-derivative (PID) controller that continuously fine-tunes the stimulation output (Wang et al., 2025). Recent computational modeling has integrated PID control with neural network optimization specifically for the basal ganglia network in Parkinson’s disease, demonstrating enhanced suppression of pathological beta oscillations compared to conventional PID or open-loop strategies (Zhang H. et al., 2025).

### 2.3 Stimulation and intervention

Based on the decision from the control algorithm, a tailored intervention is delivered to modulate neural activity (Davidson et al., 2024). The array of stimulation modalities is vast (Wang, 2024; Myers and Hossain, 2024) and can be broadly classified based on their level of invasiveness (Batsikadze et al., 2013; Rosenthal et al., 2024).

## 3 Enabling technologies

The conceptual framework of sensing, decoding, and stimulation outlined above defines the functional requirements of a closed-loop system. Translating these requirements into reliable, long-term operable devices, however, depends critically on parallel advances in hardware and computational algorithms. The following section reviews these enabling technologies—ranging from novel neural interfaces and wireless implantable systems to artificial-intelligence-driven decoding architectures—which collectively determine the performance ceiling of current and future closed-loop neuromodulation platforms.

### 3.1 Advanced sensing and materials

The development of fully implantable, wireless, and battery-less systems is a major frontier, freeing subjects from cumbersome tethers and enabling long-term studies in more naturalistic environments (Yang Z. et al., 2025; Alsamri et al., 2025). Emerging endovascular BCIs, which are delivered through the cerebral vasculature, represent a minimally invasive alternative that avoids direct brain penetration and has shown initial promise in patients with Alzheimer’s disease (Sun et al., 2025). Progress in materials science is leading to novel neural interfaces that are more biocompatible and stable. For instance, soft, flexible, and conductive hydrogels can create a seamless, low-impedance interface with neural tissue, improving signal quality and reducing foreign body response (Mangano et al., 2025). For spinal cord injury repair, hydrogel-based tissue engineering has further demonstrated the ability to bridge lesion cavities and promote axonal regeneration, offering a promising complementary strategy for closed-loop neuroprosthetic integration (Shui et al., 2026). Furthermore, new modalities are expanding the toolkit. Wearable ultrasound devices offer the potential for non-invasive, targeted, and closed-loop therapy for deep tissues (Cotero et al., 2020), while advances in optical neuromodulation and wireless optoelectronics provide unparalleled spatiotemporal precision for research applications (Mickle et al., 2019).

### 3.2 AI and computational advances

Artificial intelligence is the engine driving the “smart” component of these systems. Deep learning models are becoming increasingly adept at decoding complex neural states from high-dimensional data, outperforming traditional signal processing methods (Karatum et al., 2023). However, the “black box” nature of many AI models is a concern in clinical settings. The development of explainable AI (XAI) is therefore crucial for building trust and understanding the reasoning behind a device’s therapeutic decisions (Kwon and Min, 2025; Chu et al., 2025). On the stimulation side, the integration of robotics with neuronavigation systems is enabling highly precise and automated delivery of non-invasive stimulation like TMS, compensating for head movements in real-time and ensuring consistent target engagement (Chen S. et al., 2025).

## 4 Technical classification

Building upon these generic hardware and algorithmic building blocks, a diverse array of closed-loop neuromodulation systems has been developed, each tailored to specific anatomical targets and clinical scenarios. To navigate this rapidly expanding landscape, a systematic classification is required. In the following section, we categorize existing systems according to their level of invasiveness—a practical proxy for the trade-off between spatial precision, surgical risk, and clinical accessibility—while acknowledging that complementary dimensions, including energy modality and control architecture, are equally relevant for a comprehensive taxonomy.

### 4.1 Invasive techniques

These methods involve surgical implantation of devices and offer high spatial precision and the ability to target deep brain structures.

Deep Brain Stimulation (DBS): Electrodes are implanted in specific brain nuclei to deliver electrical pulses. Closed-loop or adaptive DBS (aDBS) has shown great promise in treating Parkinson’s disease by adjusting stimulation based on pathological beta-band oscillations and in depression by triggering stimulation based on mood-related biomarkers (Scangos et al., 2021).

Microelectrode Arrays: These arrays can both record and stimulate neural activity at a very fine scale, enabling high-resolution brain-computer interfaces (BCIs) and targeted interventions (Cao et al., 2026).

### 4.2 Non-invasive techniques

These methods modulate brain activity from outside the body, offering greater safety and accessibility (Chapman et al., 2023).

Transcranial Electrical Stimulation (tES): This category includes transcranial direct current stimulation (tDCS), which uses constant, low-intensity currents to modulate cortical excitability (Ren et al., 2022), and transcranial alternating current stimulation (tACS), which uses oscillating currents to entrain endogenous brain rhythms. Closed-loop tACS can synchronize stimulation with the phase of ongoing neural oscillations to maximize its effect (Pope et al., 2025).

Transcranial Magnetic Stimulation (TMS): TMS uses magnetic coils to induce electrical currents in the brain, allowing for precise, targeted stimulation. EEG-triggered TMS systems can deliver pulses at specific moments of cognitive processing or during specific phases of brain oscillations, creating a powerful tool for probing and modulating neural circuits (Chen S. et al., 2025).

Transcranial Focused Ultrasound (tFUS): An emerging technique that uses acoustic energy to non-invasively modulate activity in deep brain structures with high spatial precision. Integrating tFUS with EEG allows for real-time assessment of its effects and adaptive modulation strategies (Berényi et al., 2012). Recent longitudinal EEG-based assessments have further characterized the neuroplastic and adaptive responses to tFUS, supporting its potential for closed-loop implementation (Fountas et al., 2005).

### 4.3 Peripheral techniques

These methods target nerves outside the brain and spinal cord.

Spinal Cord Stimulation (SCS): Used primarily for chronic pain, closed-loop SCS systems measure the spinal cord’s response to stimulation (evoked compound action potentials) and adjust the output to maintain consistent therapy, regardless of the patient’s posture or activity level (Matias et al., 2019).

Optogenetic Peripheral Neuromodulation: This research technique uses light to control genetically modified neurons (Ma et al., 2020). Wireless, closed-loop systems can sense organ function (e.g., bladder distension) and deliver precise optical stimulation to peripheral nerves to correct pathological behavior in real-time (Salanova et al., 2021). The mechanistic basis of these optical approaches, including the biophysics of light-sensitive ion channels and tissue optical clearing, has been comprehensively reviewed, providing a foundational reference for designing closed-loop optogenetic systems (Meador et al., 2015).

### 4.4 Comparative summary

The three categories delineated above—invasive, non-invasive, and peripheral—are not rigidly discrete but rather represent points along a continuum of invasiveness and neural access. Invasive techniques (e.g., aDBS, microelectrode arrays) offer the highest spatial resolution and direct access to deep subcortical nuclei, yet they incur surgical risks, foreign-body responses, and long-term stability concerns that constrain their use to patients refractory to conventional therapies. Non-invasive techniques (tES, TMS, tFUS) provide superior safety and patient acceptance at the cost of lower spatial specificity and greater inter-individual response variability, making them suitable for broader applications including cognitive enhancement. Peripheral approaches (SCS, optogenetic peripheral modulation) occupy a middle ground—they are less invasive than intracranial implants but more targeted than transcranial methods, with particular advantages for pain and visceral disorders. Importantly, the choice among these modalities cannot be based on invasiveness alone; it must consider the target anatomy, the required temporal and spatial precision, the availability of robust biomarkers, and the acceptable risk–benefit profile for each specific indication. A taxonomic overview of the three modality categories and their representative techniques is illustrated in Figure 2. A side-by-side comparison of signal-to-noise ratio, surgical risk, stability, and cost across the three modality categories is presented in Table 1. This multidimensional trade-off landscape directly informs the clinical evidence and application scope discussed in the following section.

**Figure 2:**
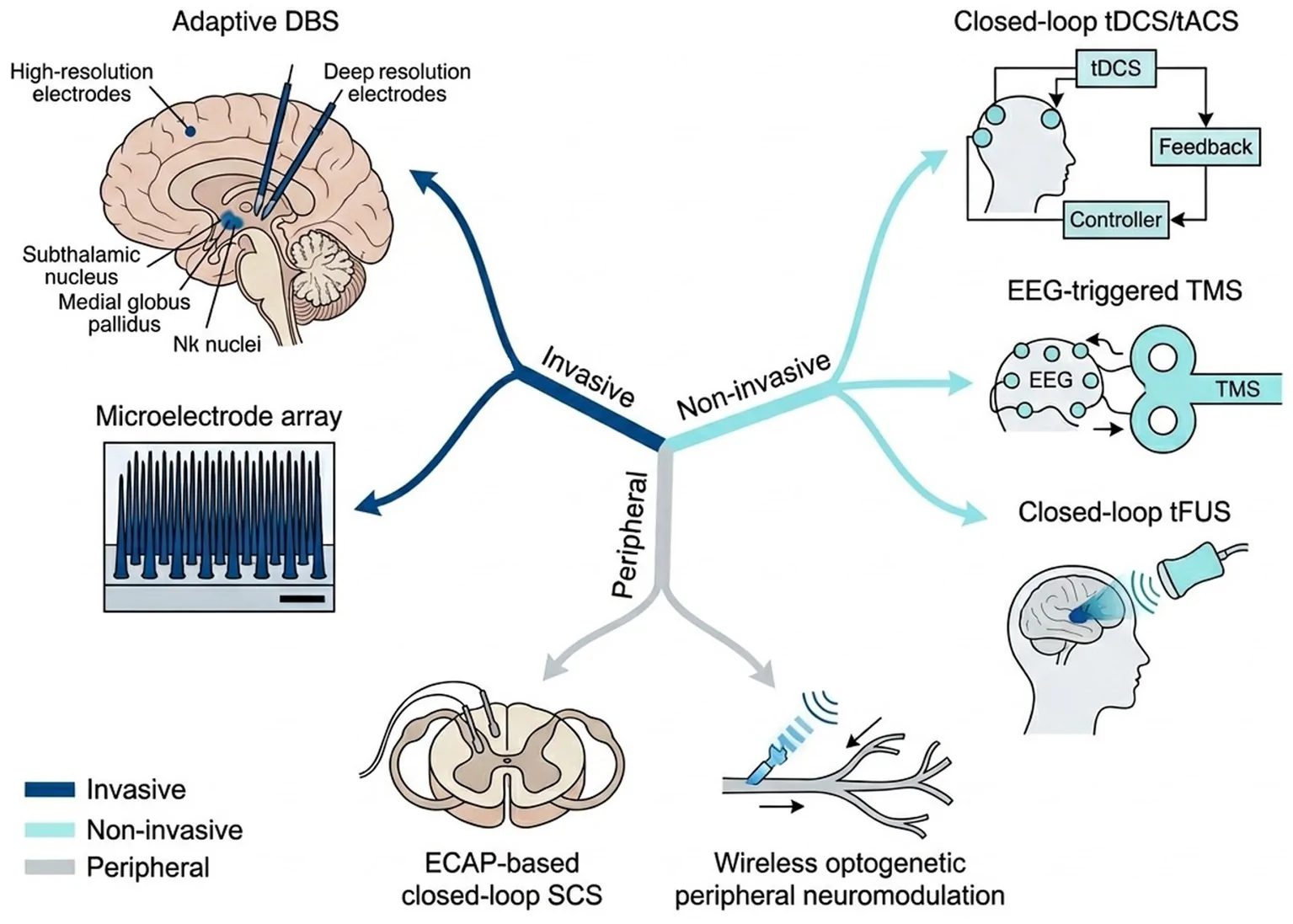
Taxonomic classification framework of closed-loop neuromodulation technologies.

**Table 1:** Comparative assessment of invasive, non-invasive, and peripheral closed-loop modalities.

Modality category	Signal-to-noise ratio	Spatial resolution	Surgical risk	Long-term device stability	Cost	Patient population	Principal side effects	Clinical accessibility
Invasive (DBS, microelectrode arrays, RNS)	High (LFP, ECoG, single-unit)	High (mm to sub-mm)	Moderate to high (craniotomy, infection, hemorrhage)	Moderate (leads can migrate; battery replacements every 2–5 years)	High	Refractory to pharmacotherapy; severe symptoms	Infection (∼4% in pediatric RNS), lead fracture, stimulation-induced dysarthria/paresthesia, cognitive side effects	Specialized centers only
Non-invasive (tES, TMS, tFUS)	Low to moderate (EEG, EMG)	Low to moderate (cm scale for tES/TMS; mm for tFUS)	Minimal (no surgery)	High (no implant, no foreign body)	Low to moderate	Broad: treatment-resistant patients, healthy individuals for cognitive enhancement	Mild (scalp discomfort, skin irritation); tFUS thermal effects under investigation	Widely available (tES/TMS); tFUS limited to research settings
Peripheral (SCS, optogenetics)	Moderate (ECAP, peripheral nerve recordings)	Moderate (nerve or spinal cord level)	Low to moderate (percutaneous or surgical lead placement)	Moderate (lead migration, fibrous encapsulation)	Moderate	Chronic pain, visceral dysfunction, peripheral motor deficits	Lead migration, infection, paresthesia (SCS); optogenetics not yet clinical	SCS widely available; optogenetics research only

SNR, signal-to-noise ratio; ECAP, evoked compound action potential; LFP, local field potential; ECoG, electrocorticography; EEG: electroencephalography; EMG, electromyography; RNS, responsive neurostimulation; DBS, deep brain stimulation; tES, transcranial electrical stimulation; tDCS, transcranial direct current stimulation; tACS, transcranial alternating current stimulation; TMS, transcranial magnetic stimulation; tFUS, transcranial focused ultrasound; SCS, spinal cord stimulation.

Existing closed-loop neuromodulation approaches are grouped into three core categories according to invasiveness: invasive, non-invasive, and peripheral modalities. Representative techniques include adaptive DBS and microelectrode arrays for invasive modulation; closed-loop tDCS/tACS, EEG-triggered TMS, and tFUS for non-invasive modulation; and ECAP-controlled closed-loop SCS and wireless optogenetic peripheral neuromodulation for peripheral intervention. While invasiveness provides a practical primary grouping, a comprehensive classification would also consider anatomical target, energy modality, and control-loop architecture (Batsikadze et al., 2013; Rosenthal et al., 2024). The color gradient from dark to light indicates decreasing invasiveness and associated surgical risk.

## 5 Clinical applications

Closed-loop neuromodulation has transitioned from a theoretical concept to a clinical reality in several domains, demonstrating its potential to transform patient care. A comparative summary of these applications, including their respective biomarkers, control strategies, and evidence levels, is provided in Table 2. The anatomical targets and feedback signals for each of these applications are schematically summarized in Figure 3.

**Table 2:** Comparative summary of closed-loop biomarkers, control strategies, and evidence levels across major clinical applications.

Indication	Sensed Signal	Biomarker	Stimulation Target	Stimulation Modality	Controller Type	Loop Latency	Developmental Stage	Principal Outcome	Evidence Level	Clinical Maturity
Focal epilepsy	ECoG	Ictal/pre-ictal pattern	Seizure focus/thalamus	RNS (electrical)	Threshold-triggered	~100–500 ms	FDA-approved (RNS System)	82% median seizure reduction at 3 years (*n* = 324); 41% with ≥90% reduction (Eliashiv et al., 2026)	I (RCT + real-world)	Established
Parkinson’s disease	LFP (STN)	Beta-band (13–30 Hz) power	STN	aDBS (electrical)	Dual-threshold adaptive	~100–300 ms	CE-marked; FDA investigational	35% greater motor improvement (MDS-UPDRS III) vs. cDBS (*n* = 5) (Cernera et al., 2025); comparable motor control with reduced side effects (*n* = 62) (Li et al., 2025)	II (multiple cohort)	Emerging
Treatment-resistant depression	Intracranial LFP	Amygdala gamma activity	VC/VS	DBS (electrical)	Personalized threshold	~200–500 ms	n-of-1/early pilot	Rapid and sustained remission in single patient (Scangos et al., 2021)	IV (n-of-1)	Exploratory
Chronic pain	ECAP	Spinal cord neural response amplitude	Dorsal column	SCS (electrical)	PID/closed-loop amplitude	~50–100 ms	FDA-approved; CE-marked	79% patients with ≥50% pain reduction at 12 months; 48% with ≥80% relief (Shariati et al., 2025)	I (RCT)	Established
Stroke rehabilitation	EEG/ECoG	Motor intent (mu/beta desync)	Cortex or peripheral nerves	BCI-triggered FES/exoskeleton	ML classifier	~200–600 ms	Research/pilot RCTs	FMA-UE improvement: mean difference of 3.26 points (95% CI: 2.73–3.78) favoring BCI (Siapas & Wilson, 1998)	II (RCT)	Emerging
Spinal cord injury	ECoG/cortical spikes	Decoded motor trajectory	Spinal cord (epidural)	Brain-spine interface (electrical)	Decoder + pattern generator	~300–500 ms	First-in-human (*n* = 1)	Standing and walking with assistance in chronic tetraplegia (Clark et al., 2024)	IV (single case)	Exploratory

Evidence level: I, meta-analysis/randomized controlled trial; II, controlled trial without randomization; III, observational study; IV, case series/n-of-1. Clinical maturity: Established, routine clinical use; Emerging, multiple ongoing trials; Exploratory, early-phase or proof-of-concept.

**Figure 3:**
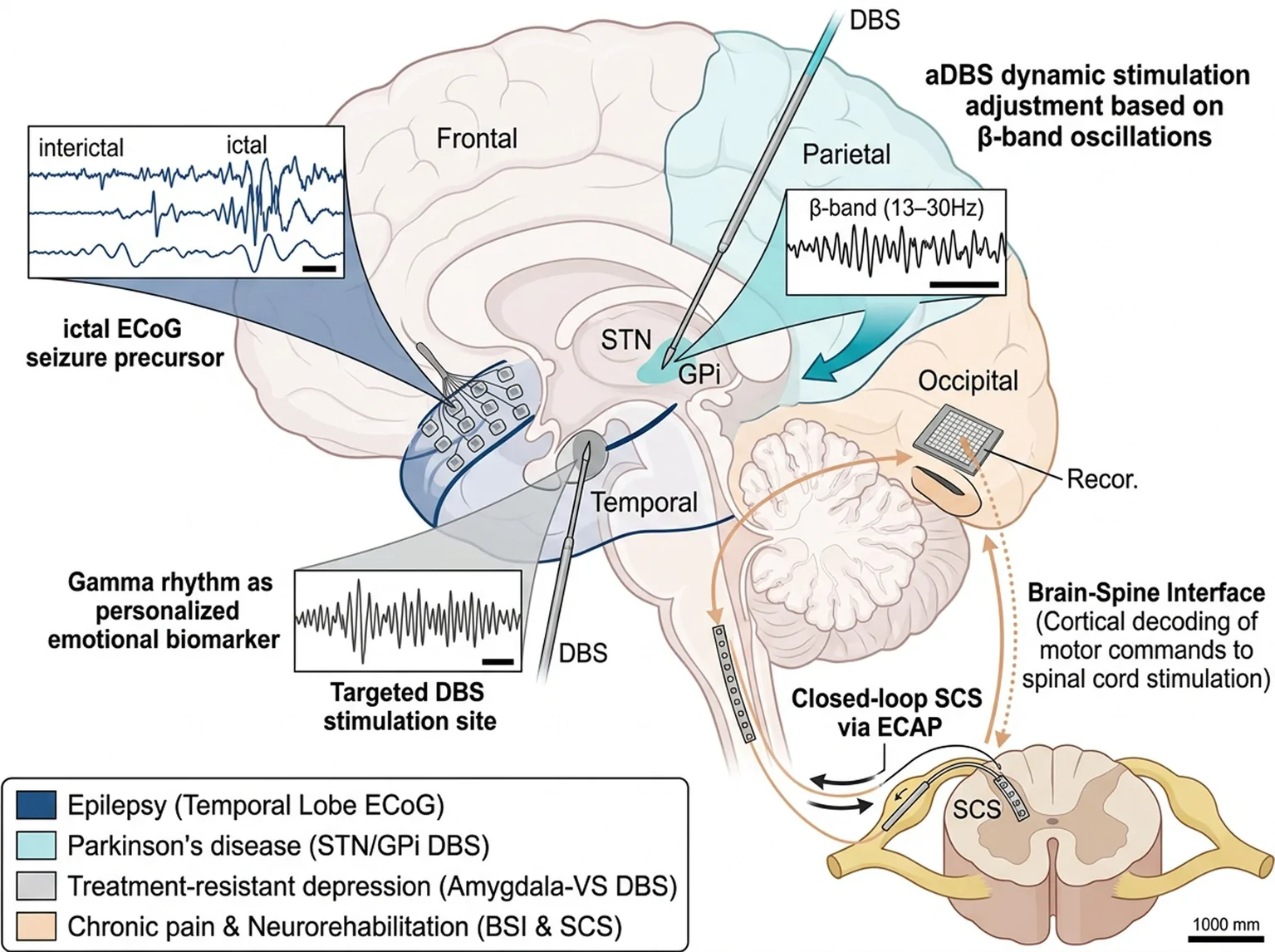
Representative clinical applications of closed-loop neuromodulation across neurological, psychiatric, and rehabilitative disorders.

Shaded boxes indicate applications with established clinical trial evidence (e.g., responsive neurostimulation for epilepsy, adaptive DBS for Parkinson’s disease), whereas dashed borders indicate exploratory or early-phase investigations (e.g., personalized DBS for depression, brain–spine interfaces for spinal cord injury). This schematic summarizes the anatomical targets and feedback signals used in each application, highlighting the varying levels of clinical validation across different indications.

### 5.1 Neurological disorders

#### 5.1.1 Epilepsy

Responsive neurostimulation (RNS) was one of the first and most successful applications of closed-loop neuromodulation (Englot et al., 2017; Ikegaya, 2025). In these systems, the device continuously monitors electrocorticographic (ECoG) activity to detect the precursors of a seizure (Yang C. et al., 2025; Morello et al., 2020). Once a specific electrographic signature is identified, the device delivers brief pulses of electrical stimulation to the seizure focus, thereby disrupting pathological activity and preventing clinical seizure manifestation (Yang C. et al., 2025; Morello et al., 2020). Clinical studies have demonstrated significant reductions in seizure frequency (Pahwa et al., 2006; Putzke et al., 2003) and improvements in quality of life among patients with drug-resistant focal epilepsy (Eliashiv et al., 2026; Munhoz et al., 2026; Piña-Fuentes et al., 2019; Liu et al., 2025).

#### 5.1.2 Movement disorders

In Parkinson’s disease, conventional open-loop DBS is highly effective for motor symptoms (Jiang et al., 2025) but can cause side effects like dysarthria (Bahadori-Jahromi et al., 2022) and paresthesia (Cernera et al., 2025; Dixon et al., 2026). Adaptive DBS (aDBS) aims to improve on this by using a biomarker, typically the power of beta-band (13–30 Hz) oscillations in the subthalamic nucleus or globus pallidus, to control stimulation (Li et al., 2025; Little et al., 2016a). Since elevated beta power is correlated with motor impairment (specifically bradykinesia and rigidity) (Little et al., 2016b; Emura et al., 2026), the aDBS system increases stimulation when beta power is high and reduces it when it is low (e.g., during voluntary movement) (Rogers et al., 2025). Studies have shown that aDBS achieves equivalent or superior motor symptom control compared to conventional DBS (Nijhuis et al., 2024; Green et al., 2025; Eisendrath et al., 2016), but with a significant reduction in total stimulation time and energy delivery, thereby reducing side effects (Fagiolini and Kupfer, 2003; Lucido and Dunlop, 2025) and potentially extending battery life (Xu et al., 2025).

#### 5.1.3 Chronic pain

The experience of chronic pain is not static (Davidson et al., 2023), and the efficacy of open-loop spinal cord stimulation can be compromised by changes in body position, which alter the distance between the electrodes and the spinal cord. Closed-loop SCS addresses this by monitoring the neurophysiological response of the spinal cord to each stimulation pulse (Berking et al., 2008). It uses this feedback to automatically adjust the stimulation intensity to maintain a consistent level of neural activation, providing more stable and effective pain relief across different activities and postures (Nakano et al., 2025; Zhang Q. et al., 2025). This technology represents a significant shift toward personalized pain management, integrating real-time physiological biomarkers to optimize therapy (Grahn et al., 2014; Shiferaw et al., 2025; Shariati et al., 2025).

### 5.2 Psychiatric disorders

Treatment-resistant depression (TRD) is a major clinical challenge (Tse et al., 2007; Klinzing et al., 2019; Stickgold, 2005; Walker and Stickgold, 2004). A landmark n-of-1 study demonstrated the profound potential of personalized, closed-loop DBS for this condition (Scangos et al., 2021). Researchers first used intracranial electrophysiology to identify a patient-specific neural biomarker of symptom severity—in this case, gamma-band activity in the amygdala. They then implanted a chronic sensing and stimulation device that delivered targeted stimulation to a different brain region (the ventral capsule/ventral striatum) whenever this biomarker was detected. This on-demand, biomarker-driven therapy resulted in a rapid and sustained remission of depressive symptoms. This work highlights the power of identifying personalized biomarkers of mood state to guide therapy, a crucial step for developing effective closed-loop psychiatric interventions (Gais and Born, 2004). The principles of emotion regulation targeted by these systems are also central to psychotherapeutic approaches, suggesting a potential synergy between technological and psychological interventions (Rasch et al., 2007).

### 5.3 Neurorehabilitation

#### 5.3.1 Stroke and brain injury

Recovery after a stroke or traumatic brain injury (TBI) depends on harnessing neuroplasticity to reorganize brain circuits (Cramer et al., 2011). Closed-loop BCIs are a promising tool to facilitate this process (Shin and Jadhav, 2024). These systems can detect the neural signature of motor intent (i.e., the user trying to move a paralyzed limb) from EEG or ECoG signals. This decoded intent can then trigger a therapeutic intervention, such as functional electrical stimulation (FES) of the muscles or the movement of a robotic exoskeleton. This closes the sensorimotor loop, providing immediate, contingent feedback that reinforces the relevant neural pathways and promotes functional recovery more effectively than conventional therapy (Siapas and Wilson, 1998).

#### 5.3.2 Spinal cord injury

For individuals with severe paralysis due to spinal cord injury, closed-loop systems are enabling unprecedented restoration of function (Jourde et al., 2025). “Brain-spine interfaces” record motor commands from the brain’s cortex, decode the intended movement, and translate these signals into patterns of electrical stimulation delivered to the spinal cord below the site of injury. This neuroprosthetic bridge can re-establish communication between the brain and the legs, allowing individuals to stand and walk again with assistance (Ngo et al., 2013; Clark et al., 2024).

### 5.4 Section summary

The clinical evidence reviewed above establishes closed-loop neuromodulation as a therapeutically viable strategy across a range of neurological and psychiatric conditions. Epilepsy (RNS) and chronic pain (closed-loop SCS) are supported by the strongest evidence, with regulatory approvals and routine clinical use. Parkinson’s disease (aDBS) and stroke rehabilitation (BCI-triggered FES) are at an intermediate stage, with multiple controlled trials demonstrating efficacy but still awaiting broader adoption. Treatment-resistant depression and spinal cord injury (brain-spine interfaces) remain exploratory, with evidence derived primarily from n-of-1 or small case series. Common to all applications is the critical dependence on robust, stable biomarkers—a recurring theme that will be examined in depth in the translational barriers section below. The gap between established and exploratory indications underscores the need for larger, multi-center trials and standardized biomarker validation protocols.

## 6 Cognitive enhancement in healthy populations

Beyond treating disease, closed-loop neuromodulation holds the potential to augment cognitive functions in healthy individuals, a field with profound implications for education, high-performance professions, and everyday life (Keeble et al., 2025). The enabling technologies that support these applications, along with their translational prospects, are illustrated in Figure 4.

**Figure 4:**
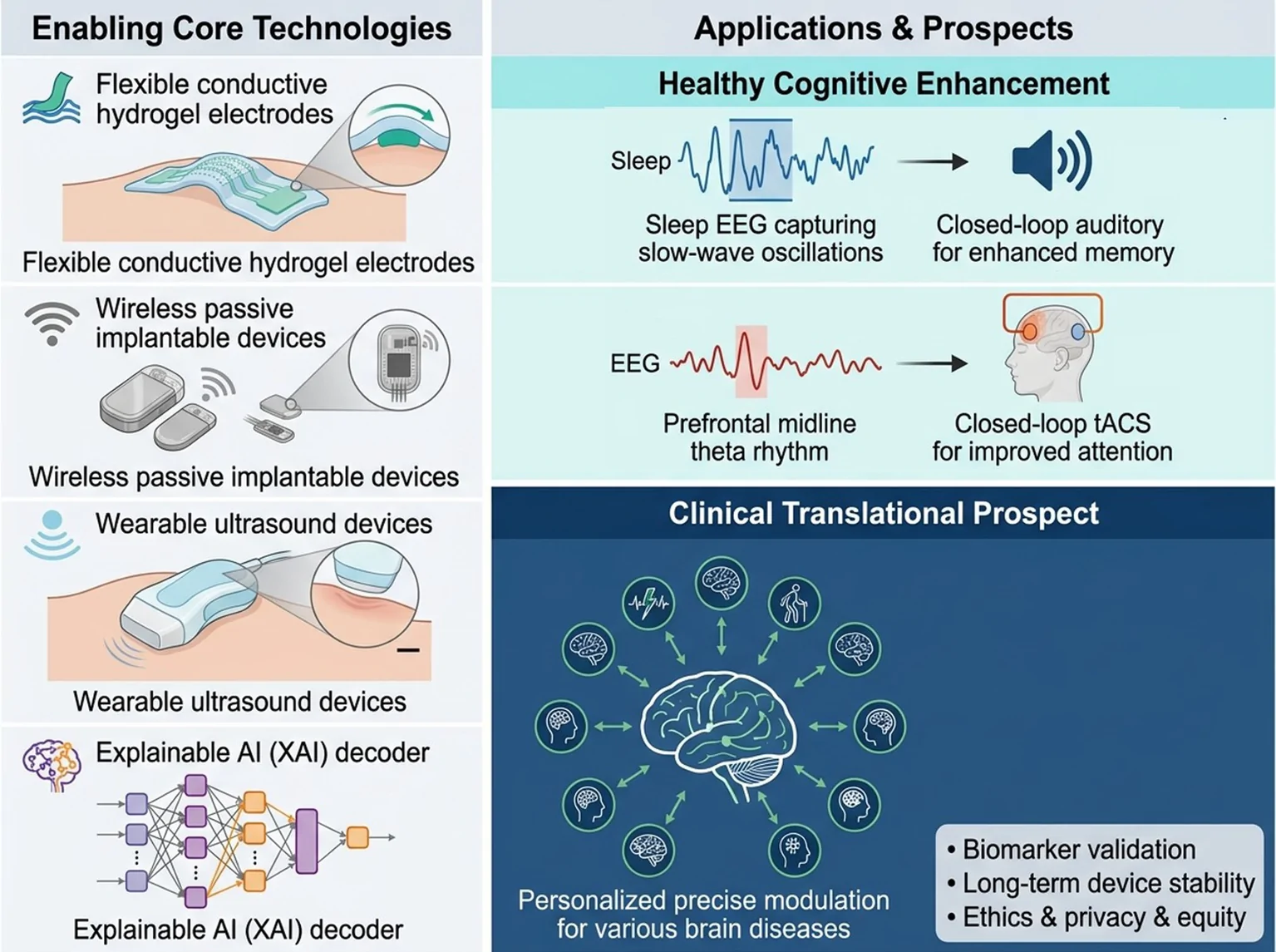
Enabling foundational technologies and dual translational prospects of closed-loop neuromodulation.

Left panel: key hardware and algorithmic advances, including biocompatible hydrogel neural electrodes, battery-free wireless implantable devices, wearable ultrasound equipment, and explainable artificial intelligence (XAI) decoding systems. Right panel: dual application streams—non-invasive closed-loop interventions for enhancing memory consolidation and attentional function in healthy individuals, and personalized precision therapy for intractable brain disorders. Marginal text boxes list major translational barriers (biomarker validation, device durability, and ethical governance). The layout emphasizes that hardware innovation and algorithmic interpretability are equally critical for responsible clinical translation.

### 6.1 Learning and memory

Memory is not formed instantaneously but is consolidated over time (Krueger et al., 2024), particularly during sleep (Coffman et al., 2014; Roediger and Butler, 2011; Theis et al., 2025). Slow-wave sleep (SWS) is critical for this process (Larsen et al., 2008; Friedman and Miyake, 2017), characterized by coordinated neural oscillations including slow oscillations, sleep spindles, and hippocampal ripples (Jurado and Rosselli, 2007; Verbruggen and Logan, 2008). Closed-loop systems can enhance memory consolidation by detecting these key oscillatory events in real-time via EEG and delivering precisely timed interventions (Enriquez-Geppert et al., 2014). For example, auditory tones delivered in phase with ongoing slow oscillations have been shown to boost these rhythms (Magosso et al., 2021) and improve subsequent memory recall (McDonnell and Strayer, 2024; Tsujimoto et al., 2006; Jamal et al., 2025). Similarly, tES or TMS can be triggered by specific EEG signatures of learning to selectively enhance synaptic plasticity at opportune moments (Lorenzetti et al., 2018), potentially accelerating skill acquisition and knowledge retention (Jiang et al., 2022). This aligns with cognitive science principles like test-enhanced learning, where active retrieval strengthens memory (Goebel et al., 1915); neuromodulation could potentially amplify these effects (Boekaerts et al., 2000; Adame et al., 2026).

### 6.2 Attention and cognitive control

Executive functions like attention and cognitive control are crucial for goal-directed behavior (Friedman and Miyake, 2017; Jurado and Rosselli, 2007; Verbruggen and Logan, 2008). These functions are associated with specific neural oscillations, such as frontal midline theta (4–8 Hz) rhythms (Enriquez-Geppert et al., 2014; Magosso et al., 2021; McDonnell and Strayer, 2024; Tsujimoto et al., 2006). Closed-loop tACS systems are being developed to monitor theta activity and deliver stimulation that amplifies these rhythms when a user needs to engage in a demanding cognitive task, potentially improving focus and reducing errors (Jamal et al., 2025; Lorenzetti et al., 2018; Jiang et al., 2022). Another approach is real-time neurofeedback, where individuals learn to voluntarily modulate their own brain activity (Goebel et al., 1915; Boekaerts et al., 2000). In a closed-loop neurofeedback system, a person’s EEG signals are translated into a sensory cue (e.g., a sound or visual display) (Adame et al., 2026) and they learn to alter this cue by changing their mental state (Liu, 2025). This form of training can enhance skills of self-regulation, which are fundamental to cognitive and emotional well-being (Theis et al., 2025; Larsen et al., 2008; Mitsea et al., 2024).

### 6.3 Section summary

Cognitive enhancement via closed-loop neuromodulation remains an emerging frontier with promising preliminary evidence. The most compelling data exist for memory consolidation during sleep, where closed-loop auditory stimulation has been replicated across multiple laboratories. Attention and cognitive control studies are fewer in number and more heterogeneous in design, but early results with closed-loop tACS and neurofeedback are encouraging. Key unresolved questions include the durability of enhancement effects, the optimal stimulation parameters for different cognitive domains, and the potential for individual differences in responsiveness. Moreover, the translation of these findings from controlled laboratory settings to real-world environments requires the development of portable, robust, and user-friendly closed-loop devices. Ethical considerations surrounding non-therapeutic cognitive enhancement—particularly equity of access and the risk of coercion—must be addressed proactively as the technology matures.

## 7 Translational barriers and ethical challenges

Despite the promising findings in both therapeutic and cognitive-enhancement applications, the widespread clinical and societal deployment of closed-loop neuromodulation faces formidable obstacles. These challenges are not merely technical—such as biomarker instability and hardware limitations—but also extend to profound ethical and regulatory questions concerning autonomy, privacy, and equity. The following chapter critically examines these translational barriers, moving beyond qualitative acknowledgment to a structured analysis of specific constraints and potential mitigation strategies.

### 7.1 Biomarker validation and stability

A primary technical challenge is the identification and validation of robust biomarkers. An effective control variable must be both sensitive and specific to the clinical or cognitive state of interest, and it must be stable over long periods. However, several fundamental issues complicate this task.

First, the specificity of many candidate biomarkers remains uncertain. Beta-band (13–30 Hz) oscillations in the subthalamic nucleus, for instance, correlate with bradykinesia and rigidity in Parkinson’s disease but are also modulated by movement, attention, and even arousal—raising the risk of false-positive stimulation triggers. A subset of Parkinson’s patients lack stable beta rhythm signals altogether, limiting the generalizability of beta-based aDBS. Second, causality is often unclear: while elevated beta power correlates with motor impairment, it remains debated whether it is a direct driver of pathology or an epiphenomenon. Interventions targeting a non-causal biomarker may achieve closed-loop control in the mathematical sense but fail to produce meaningful therapeutic benefit. Third, temporal drift—the gradual change of signal characteristics over months to years due to disease progression, neuroplasticity, or electrode-tissue interface evolution—can render a once-valid biomarker ineffective over time. Fourth, artifacts from movement, muscle activity, or environmental noise frequently contaminate neural recordings, particularly in non-invasive EEG-based systems, and can lead to inappropriate stimulation delivery if not adequately filtered.

To address these challenges, multimodal signal fusion frameworks—combining EEG, LFP, fMRI, and peripheral physiological signals—are being explored to improve specificity and reduce false positives. However, the optimal fusion strategies and their computational costs remain active areas of investigation.

### 7.2 Hardware and algorithmic constraints

The practical deployment of closed-loop neuromodulation systems is constrained by multiple hardware limitations that are often underappreciated in conceptual reviews.

Power consumption and heat dissipation remain critical barriers for fully implantable devices. Real-time neural decoding and adaptive stimulation algorithms require significant computational resources, which in turn increase power draw and generate heat that must be safely dissipated within the cranium or subcutaneous space. Battery life, currently 2–5 years for most implantable DBS systems, imposes a ceiling on the complexity of on-board processing. Memory capacity limits the storage of long-term neural recordings needed for personalized algorithm calibration. Computational latency—the delay between signal acquisition and stimulation delivery—is a performance determinant that can vary from 50 ms in simple threshold-triggered SCS to over 500 ms in complex BCI decoding pipelines; for applications such as seizure termination, even 200 ms may be clinically significant. Wireless bandwidth constraints limit the transmission of raw high-channel-count neural data to external processors, necessitating on-device feature extraction that is both power-hungry and technically challenging. Data security concerns arise when devices are connected to external networks for algorithm updates or remote monitoring, creating potential vectors for unauthorized access to sensitive neural data.

Progress in low-power electronics, edge computing, and energy-harvesting technologies is gradually alleviating some of these constraints, but a fundamental trade-off between functionality and implantability persists.

### 7.3 Ethical and regulatory frameworks

Perhaps most importantly, the advent of intelligent devices that can read and write to the brain raises profound ethical questions that extend beyond the standard concerns of medical device regulation.

Personal agency and identity are at the forefront of these concerns. As closed-loop systems become more autonomous—making real-time decisions about when and how much to stimulate—they challenge traditional notions of individual control over one’s own mental and emotional states (Lavazza et al., 2025). Continuous mood regulation, for example, could alter a patient’s sense of self or emotional authenticity. Who is responsible when an autonomous device makes a suboptimal therapeutic decision—the prescribing clinician, the manufacturer, or the algorithm designer? (Marques et al., 2024). These questions of accountability are not merely philosophical; they have direct implications for liability, insurance, and regulatory oversight.

Neural data privacy is another critical concern. The sensitive neural data collected by these devices could theoretically reveal information about an individual’s thoughts, emotions, preferences, or even unconscious biases. Robust frameworks for data governance—including encryption, access control, and transparent data-use policies—are essential to protect patients from misuse (Boonstra, 2025). Emerging international initiatives, including the “neurorights” movement in Chile and other jurisdictions, have proposed constitutional protections for mental privacy and cognitive liberty (Trabucco, 2023), though concrete implementation remains in early stages.

Equity and accessibility pose additional societal challenges. The high cost and specialized expertise required for invasive closed-loop systems could exacerbate existing healthcare disparities. The potential for non-therapeutic cognitive enhancement—if realized—could create a societal divide between the “enhanced” and “unenhanced,” raising questions of fairness in education, employment, and other competitive domains (Ridolfi and Santos, 2025). Proactive policy discussions, involving neuroscientists, clinicians, ethicists, patients, and the public, are needed to ensure that these technologies are developed and deployed in a manner that promotes rather than undermines social equity.

### 7.4 Section summary

The translational barriers to closed-loop neuromodulation are substantial and span technical, clinical, and ethical domains. Biomarker instability, hardware constraints, and algorithmic challenges require sustained engineering innovation; the limited generalizability of current evidence demands larger, more rigorous clinical trials; and the profound ethical implications call for proactive governance frameworks. Importantly, these challenges are not insurmountable—but they do require an integrated, interdisciplinary approach that engages stakeholders across neuroscience, engineering, clinical medicine, law, and ethics. Table 3 summarizes the key unresolved scientific and translational questions that, in our view, warrant focused investigation over the next 3–5 years.

**Table 3:** Priority unresolved scientific and translational questions for the next 3–5 years.

Domain	Key unresolved questions
Biomarker	How to identify stable, causal, and generalizable biomarkers across individuals and over years? How to optimally fuse multimodal signals (EEG, LFP, fMRI, peripheral) to reduce false positives?
Hardware	How to balance power consumption, heat dissipation, wireless bandwidth, and long-term (>10 years) biocompatibility in fully implantable devices?
Algorithm/control	How to integrate explainable AI into real-time clinical decisions without sacrificing accuracy? Which control strategies (MPC, RL, adaptive PID) suit which temporal dynamics?
Clinical translation	What efficacy thresholds and safety benchmarks justify regulatory approval? How to design trials that adequately test superiority over open-loop systems?
Ethics and society	How to protect neural data privacy while enabling algorithm updates? How to allocate responsibility for autonomous device decisions? How to ensure equitable access to cognitive enhancement?

## 8 Conclusion

Closed-loop neuromodulation represents a fundamental evolution in our ability to interact with the nervous system. By moving from a monologue of open-loop stimulation to a real-time, adaptive dialogue, this paradigm offers a pathway to truly personalized and precise interventions. The progress to date across a spectrum of clinical applications—from epilepsy and depression to chronic pain and motor rehabilitation—demonstrates its therapeutic potential, while emerging applications in cognitive enhancement open new frontiers for understanding and augmenting human performance (Joseph, 2025).

The future of this field depends on continued innovation in wireless biocompatible hardware, explainable AI algorithms, and standardized biomarker validation. However, as we develop more powerful tools to modulate the brain, we must proceed with caution. A proactive, inclusive dialogue among scientists, clinicians, ethicists, patients, and the public is essential to navigate the complex ethical landscape. By embracing this interdisciplinary and ethically grounded approach, closed-loop neuromodulation can fulfill its promise—not only to improve the treatment of brain disorders but also to deepen our fundamental understanding of the human mind (Morente Parra, 2025; Gutruf et al., 2024).
